# Brain activity as a candidate biomarker for personalised caffeine treatment in premature neonates

**DOI:** 10.3389/fped.2026.1792897

**Published:** 2026-05-04

**Authors:** Fatima Usman, Coen S. Zandvoort, Shellie Robinson, Mariska Peck, Maria M. Cobo, Tricia Adjei, Luke Baxter, Ria Evans Fry, Annalisa G. V. Hauck, Richard Rogers, Gabriela Schmidt Mellado, Alexandra Scrivens, Marianne van der Vaart, Maarten De Vos, Eleri Adams, John van den Anker, Caroline Hartley

**Affiliations:** 1 Department of Paediatrics, University of Oxford, Oxford, United Kingdom; 2 Colegio de Ciencias Biologicas y Ambientales, Universidad San Francisco de Quito USFQ, Quito, Ecuador; 3 Nuffield Division of Anaesthetics, John Radcliffe Hospital, Oxford University Hospitals NHS Foundation Trust, Oxford, United Kingdom; 4 Department of Electrical Engineering (ESAT), STADIUS Center for Dynamical Systems, Signal Processing and Data Analytics, KU Leuven, Leuven, Belgium; 5 Department of Development and Regeneration, University Hospitals Leuven, Child Neurology, KU Leuven, Leuven, Belgium; 6 Newborn Care Unit, John Radcliffe Hospital, Oxford University Hospitals NHS Foundation Trust, Oxford, United Kingdom; 7 Division of Clinical Pharmacology, Children’s National Hospital, Washington DC, United States

**Keywords:** apnoea of prematurity, brain age, caffeine, EEG, neonate

## Abstract

**Background:**

Caffeine is one of the most frequently administered medicines in neonatology—prescribed for the management of apnoea of prematurity, to aid extubation and increasingly for conditions such as bronchopulmonary dysplasia. Caffeine guidelines for the management of apnoea of prematurity indicate use based on the age of the infant, but this does not account for individual variation in apnoea rate. Consequently, infants may risk caffeine undertreatment or adverse events due to over-exposure. Apnoea in preterm infants is related to nervous system immaturity, hence, as an essential first step to assess whether brain activity may be a useful biomarker for caffeine treatment, we tested the hypothesis that apnoea rate is related to brain activity.

**Methods:**

In this single-centre prospective observational cohort study, we simultaneously recorded brain activity using electroencephalography (EEG) and respiration using impedance pneumography in 74 infants aged 31–36 weeks postmenstrual age (PMA) on 138 separate occasions. The primary outcome was the association between apnoea rate and brain age gap (defined as the difference between the infant's brain age and their PMA; brain age is calculated from brain activity using a deep learning algorithm). In an exploratory sub-study, we compared the apnoea and desaturation rate in the 7 days after infants stopped caffeine treatment, between those infants with immature and mature brain activity.

**Results:**

We demonstrate that apnoea rate in moderate/late preterm infants is dependent on brain age gap (p:0.024; *β* [95% CI]:−0.22 [−0.41 to −0.03]). In contrast, apnoea rate was not correlated with PMA (p:0.58; *β* [95% CI]:−0.04 [−0.16 to 0.09]). In the exploratory sub-study, we find that when caffeine is discontinued, infants with immature brain activity have more frequent apnoeas and desaturations compared with those with more mature brain function.

**Conclusions:**

These findings provide initial evidence to indicate that brain age is a candidate biomarker for personalised caffeine treatment in preterm infants.

## Background

Apnoea of prematurity (AOP) is a developmental respiratory disorder affecting more than half of the 15 million infants born prematurely each year worldwide (1, 2). Apnoea is a breathing cessation of variable duration which can be life-threatening and may impact long-term neurodevelopmental outcomes (3, 4), so it is essential to optimise treatment. AOP is a consequence of nervous system and pulmonary immaturity of preterm infants (5) and will usually resolve by late prematurity (6).

**Figure 1:**
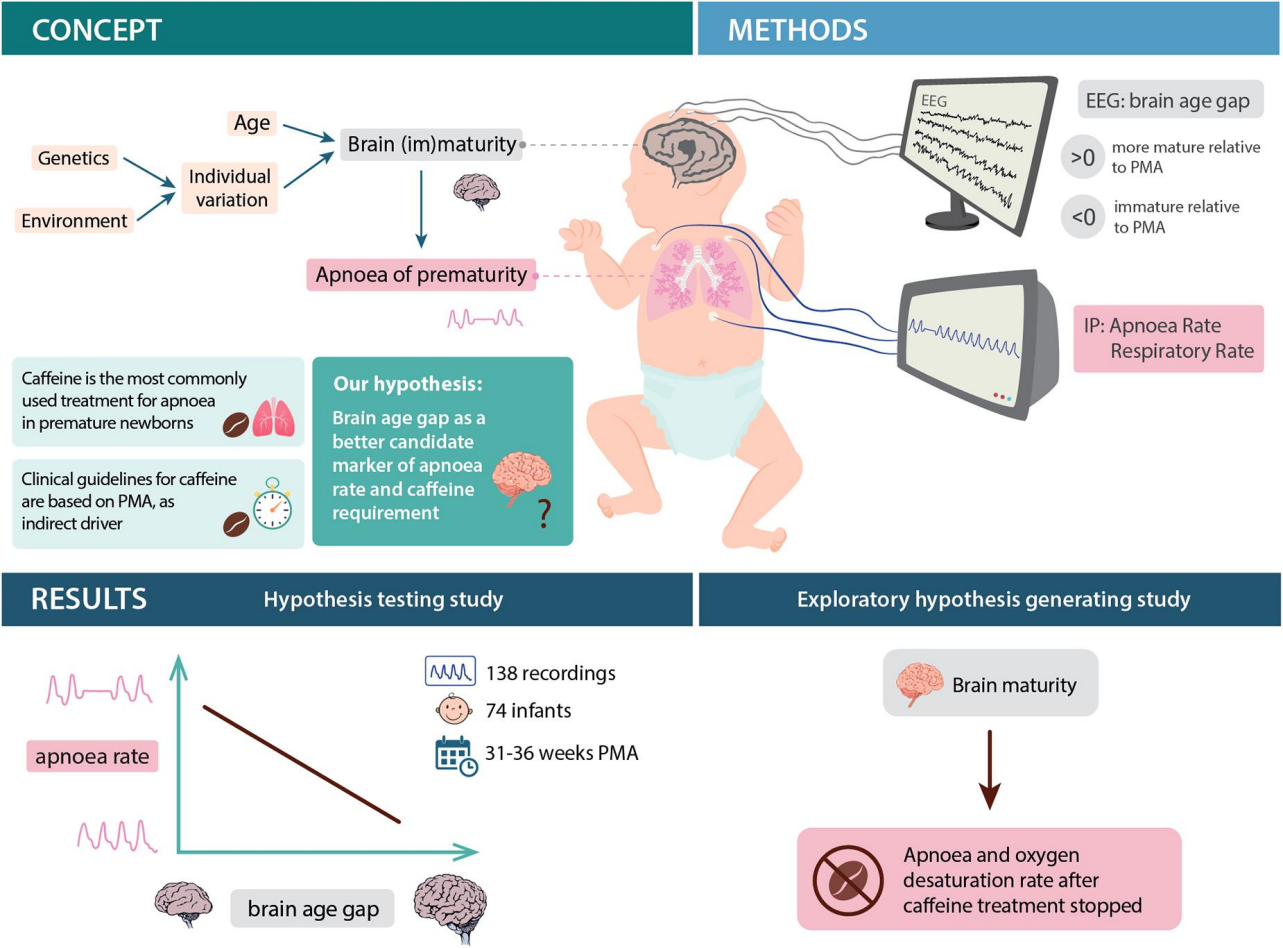
Schematic of study concept, design and main results. EEG: electroencephalography, IP: impedance pneumography, PMA: postmenstrual age. Note that other factors, such as infection, can lead to apnoea in premature infants; however, here we focus on the relationship between apnoea and brain development.

Caffeine is the primary pharmacological treatment for AOP, reducing apnoea rates, duration of ventilatory support and extubation failure, and improving neurodevelopmental outcomes (7). Clinical guidelines for the treatment and prevention of AOP indicate caffeine requirement based on gestational age or weight of an infant (8). For example, the UK's National Institute for Health and Care Excellence (NICE) guidelines suggest to “consider stopping caffeine citrate at 33–35 weeks” corrected gestational age if the baby is clinically stable' (9). Whilst this does consider the stability of the baby, it nevertheless also relates to age, precluding stopping treatment before 33 weeks. Prescription based on age does not directly relate to the underlying cause of apnoea (i.e., immaturity of the brain and pulmonary systems) (Figure 1). This may expose some infants to caffeine-related adverse effects, such as tachycardia, reflux and feeding intolerance, or rarely seizures (10, 11) if, for example, they receive caffeine unnecessarily. Moreover, some infants discontinue treatment too early and experience substantial apnoeas necessitating recommencement of treatment (12). An individualised approach is needed (8, 13) and can be achieved through identification of biomarkers for caffeine requirement. Currently, no such biomarkers have been identified.

Given the direct link between brain development and apnoea, here we propose brain activity, and more specifically, a metric derived from brain activity known as the brain age gap, as a candidate biomarker (Figure 1). We define brain age gap as the difference between an infant's “brain age” and their postmenstrual age (PMA) (14, 15). Thus, the brain age gap is a marker of whether an individual's brain activity is more or less mature relative to their PMA. Brain age can be assessed using electroencephalography (EEG), combined with recently developed and validated machine learning models (16–19). These models capitalise on the fact that the EEG changes rapidly in preterm infants, both in background activity (20) and sensory-evoked responses (21–23) to detect maturity-related differences in brain activity. Very immature brain activity relative to PMA may be associated with poorer later life neurodevelopmental outcomes (16, 17), yet there will also be normal variation in brain age gap—just as older children learn to walk and talk at different ages, brain developmental trajectories exhibit individual variation modulated by factors such as genetics and the environment (24, 25). We postulate that this individual variation in brain age gap (i.e., individual variation in EEG activity) will explain variation in apnoea rate and consequently could be used to guide treatment with caffeine.

There is limited evidence to date to suggest that brain activity in human preterm infants is related to apnoea rate: Henderson-Smart and colleagues (26) demonstrated prolonged auditory brainstem conduction times in infants with AOP. Moreover, we recently demonstrated that cortical brain activity (most likely originating from cortical motor areas) is involved in the regulation of breathing in infants and that infants with stronger “coupling” between cortical and respiratory activity had lower incidences of apnoea (27). In the present study, we hypothesised that, in late preterm infants, apnoea rate is related to brain age gap and that this relationship is stronger than the relationship between apnoea rate and PMA. Indeed, demonstrating this is an essential first step to suggest that brain age gap may be a useful candidate biomarker for caffeine treatment; if this were not the case, then treatment based on an alternative biomarker would be a better approach. For this study, we focused on moderate-to-late preterm infants from 31 to 36 weeks PMA to investigate whether brain age gap can be used to assess caffeine requirement. It is possible that some of even the youngest infants in this age range will not need to start caffeine treatment and that brain age gap could be used to guide this decision. Moreover, brain age gap may be a candidate biomarker to indicate when to discontinue treatment in preterm babies receiving caffeine, which is an important step in their care, could expedite discharge and shift the balance in an individual from potential adverse effects and towards benefit.

## Methods

### Study design, setting and participants

This was a prospective cohort study conducted at the Newborn Care Unit of the John Radcliffe Hospital, Oxford University Hospitals NHS Foundation Trust, Oxford, UK between 2019 and 2023. Clinically stable preterm infants aged between 31 weeks + 0 days up to 36 weeks+6 days PMA at the time of test occasion were eligible to be included in the study. Infants were excluded if they had grade III or IV intraventricular haemorrhage, hypoxic ischaemic encephalopathy, congenital malformations, were on mechanical ventilation, had a significant respiratory problem (e.g., pneumonia, persistent pulmonary hypertension, pneumothorax), were receiving opioid analgesics at the time of study, or if there was a history of maternal substance misuse during pregnancy. These conditions are known to impact brain activity and may interfere with the interpretation of the EEG by acting as confounders. Previous studies on brain age models utilised similar inclusion and exclusion criteria (16, 17).

The study was approved by the National Research Ethics Service (references: 19/LO/1085, 12/SC/0447). Eligible families were given verbal and written information about the study, and written parental consent was signed before inclusion in the study. The study conformed to the standards set by the Declaration of Helsinki and Good Clinical Practice.

As the study was exploratory a sample size calculation was not possible, and a heuristic approach was used. An initial total of 76 infants were studied on 141 separate test occasions; 53 infants were included in one to three test occasions, 23 infants took part in the ongoing *Breathing and Brain Development* study (28) where their EEG is recorded approximately once a week and their vital signs recorded continuously whilst on the Newborn Care Unit [included on average on 3 ± 1 (mean ± SD) test occasions]. During each test occasion (which aimed to last ∼2 h), simultaneous EEG and vital signs (respiration, heart rate and oxygen saturation) were recorded. Three test occasions were excluded due to very short recordings of inter-breath intervals (see “computing respiratory and apnoea rate”), leaving a total of 74 infants studied on 138 test occasions included in the analysis.

### Data acquisition

#### Vital signs recordings

Each infant had continuous vital signs monitoring as the standard of care. Vital signs were monitored using Phillips IntelliVue MX800 or MX750 monitors and data were continuously downloaded from the vital signs monitor using an electronic data capture software (iXtrend, ixitos, Germany). For infants in the *Breathing and Brain Development* study, vital signs were downloaded continuously throughout the infant's hospital stay; the recordings from the day of the EEG test occasion are included in the main analysis (i.e., up to 24 h of recording). For other infants, vital signs were recorded during EEG data acquisition for an average of 1.5 h (median, range: 0.4–3.3 h).

Heart rate, oxygen saturation and respiratory rate (calculated by the monitor) were downloaded onto the laptop at a sampling rate of 0.97 Hz; the electrocardiograph (ECG, to measure heart rate) at 250 Hz and recorded with three electrodes placed on the infant’s chest; the impedance pneumography (IP, to measure respiration) at 62.5 Hz and recorded from the chest electrodes, and the photoplethysmography (PPG, to measure oxygen saturation and pulse) at 125 Hz from a probe placed on the infant's foot or hand.

#### EEG recordings

EEG was recorded from DC to 400 Hz using a SynAmps RT 64-channel headbox and amplifiers and CURRYscan7 neuroimaging suite (Compumedics Neuroscan) at a sampling rate of 2 kHz. Eight electrodes were placed at Cz, CPz, C3, C4, Oz, FCz, T3, and T4, according to the modified international 10–20 system. The ground electrode was placed at FPz and the reference electrode at Fz. Infant scalp preparation was done with NuPrep gel (D.O. Weaver and Co., Aurora, USA) to achieve impedances of 5–10 k*Ω*. A conductive paste (Elefix EEG paste, Nihon Kohden, Tokyo, Japan) was used to attach disposable Ag/AgCl cup EEG electrodes (Ambu Neuroline, Ballerup, Denmark) to the infant's scalp. At the beginning of each recording, approximately 10 background annotations were time-locked on the EEG and vital signs recordings by manually pushing a button connected to a bespoke triggering device (the PiNe box) (29) which simultaneously annotates the EEG and vital signs recordings. This enabled synchronisation of the vital signs and EEG recordings. Background annotations were performed by a researcher during a period when the infant was quietly resting in their cot/incubator. Resting-state EEG was recorded for an average of 1.7 ± 0.7 h (mean ± SD). No clinical records on the normality of the EEG were available.

#### Tactile and visual stimuli

A tendon hammer was used to lightly tap the infant's foot to elicit touch responses. At least 10 gentle touch stimuli, with an inter-stimulus interval of approximately 10 s (longer if the infant was unsettled) were applied. The tendon hammer was modified with a built-in force transducer (Brüel & Kjær, Denmark) that sends a trigger pulse at the point of stimulation (30). For visual-evoked responses, the stimulus was a flash of light presented using a Lifelines Photic Stimulator (intensity level 4, approximately 514 lumens). The Photic Stimulator was placed approximately 30 cm away from the infants, in line with their direct field of vision and a train of 10 light flashes was delivered at least 10 s apart. The tactile and visual stimuli (order pseudo-randomised) were performed either at the start or end of the EEG recording. Researchers viewed the EEG at the time of recording to ensure that stimuli were delivered during periods where the infant was settled and the EEG was free from artefacts. The stimuli were annotated simultaneously on the EEG and vital signs recordings using our custom-made time-locking device (the PiNe box) (29).

#### Recording of infant demographics and clinical details

Relevant demographic and clinical information were obtained from the medical notes of each infant and updated at each test occasion, where relevant. These included the infant's age at birth and sex, and at the time of the test occasion, the postmenstrual age, current mode of ventilation and presence of infection. Infection was defined based on the neonatal guidelines for infection treatment at the John Radcliffe Hospital as either an elevated C-reactive protein of >10 mg/L, abnormal white blood cell count (>20 × 10^9^/L or <5 × 10^9^/L), a positive blood culture; or a symptomatic infant with predisposing factors for sepsis receiving antibiotics at the time of EEG recording.

The age at which caffeine was stopped was dependent on clinical decision (clinicians were blinded to the EEG recording and EEG outcome metrics) and independent of the research study. In our unit, local guidelines indicate starting caffeine treatment in all infants born <32 weeks' gestation, aiming to start treatment within the first hour of life. They further state to “consider discontinuing caffeine between 32 and 34 weeks corrected GA if no apnoeas or bradycardias requiring stimulation” and “if still on respiratory support and/or having significant apnoeas or bradycardias, continue until 36 weeks gestation or later if beneficial”. In a recent study of a large cohort in our unit, the average PMA at which infants stopped caffeine treatment was 34 weeks (31).

### Data analysis

All analyses were performed in MATLAB (ver. 2022b; MathWorks Inc., Natick, USA). In brief, we first computed respiratory and apnoea rates from the IP signals, and brain age gap from 20 min of resting state EEG activity and sensory-evoked (visual and tactile) potentials. Next, we constructed linear (mixed-effects) models to test if the respiratory outcomes—respiratory and apnoea rates—were associated with either PMA or brain age gap. We also explored whether the PMA at which caffeine treatment is discontinued relates to brain age gap. In a final exploratory analysis to explore whether the brain age gap could be utilised as a predictive biomarker for stopping caffeine treatment, we investigated whether the rate of episodes of physiological instabilities (apnoea and oxygen desaturation rate) in the week after caffeine was stopped is related to brain age gap. Each of these analysis steps are outlined in more detail below.

#### Computing respiratory and apnoea rate

To obtain respiratory and apnoea rates, IP signals were processed using an algorithm validated for the identification of inter-breath intervals (IBIs) and apnoeas in infants (32). This identifies central apnoeas; obstructive apnoeas could not be identified as nasal air flow was not measured. Briefly, the algorithm first filters the signal to reduce the noise introduced by movements and cardiac activity. An adaptive amplitude threshold (set at 0.4 times the standard deviation of the signal over the preceding 15 breaths) is used to identify individual breaths. Finally, a support vector machine algorithm was applied to all potential episodes of apnoea [defined as IBIs longer than 15 s (33)] to remove periods of low amplitude erroneously identified as apnoeas due to noise or shallow breathing. This model was trained and validated on IP signals acquired during a clinical trial (34). The data from the clinical trial was completely independent from our current data sample. Long IBIs marked as noise/shallow breathing were discarded from further analysis. We then computed respiratory and apnoea rates estimated from all available IBIs on the day of the EEG recording, which was 8.5 ± 8.7 h in duration (mean ± SD over recordings). We computed respiratory rates as the median number of breaths per minute (i.e., 60 divided by the median IBI of the recording) and apnoea rates as the number of apnoeas [IBIs greater than 15 s (33)] per hour from all available IBIs on the day of the EEG recording. We excluded three recordings which had fewer than 100 IBIs available.

#### Estimating brain age gap from EEG activity

To obtain the brain age gap, we first computed brain age using two brain age models (16, 17). These two models estimate the functional maturation of the brain using resting state (17) and sensory-evoked EEG activity (16), respectively. These models aim to capture different developmental aspects. The resting state model is an index for the maturation of ongoing brain activity and the sensory-evoked model for the maturation of sensory pathways (Supplementary Figure S1). Whilst both models use features extracted from different neural systems, all features change in an age-related manner. Both are machine learning models which are trained to estimate the age of the infant from the EEG activity, with the aim of such models being to identify infants who deviate from normal neurodevelopment. Thus, brain age is correlated with PMA, and large differences between PMA and brain age may be indicative of abnormal outcome (16, 17). Brain age gap was defined as the mean brain age of the two models minus PMA (note, brain age gap has also been referred to in the literature as the brain age delta). Supplementary Figure S2 compares brain age and respiration/apnoea rate.

Before estimating the brain age gap, EEG data were first pre-processed using the Brainstorm (version 3) (35) and EEGLAB (version 2022.1) (36) toolboxes. EEG time series were filtered with pass-band edges at 0.1 and 30 Hz by consecutively high-pass (Hamming windowed-sinc FIR filter with a cut-off frequency at 0.05 Hz) and low-pass filtering the signals (Hamming windowed-sinc FIR filter with a cut-off frequency at 33.75 Hz).

To calculate the infant's brain age from their sensory-evoked responses (visual and tactile), we used the model developed by Zandvoort and colleagues (2024) (16). Briefly, this model capitalises on the PMA-related changes in the morphology of sensory-evoked potentials during the preterm period. The sensory-evoked data were first epoched from −0.5 to 1.0 s around the stimulus onsets for channels Oz and Cz. Epochs were baseline normalised by subtracting the mean amplitude of the time window between −0.5 and 0 s. Epochs were then visually inspected, and those with excessive amplitudes or artefacts were excluded (an average of 10% of epochs were excluded). We rejected epochs when the amplitude in the pre-stimulus window exceeded ±150 µV between −1 and 0 s relative to the stimulus onset. Individual test occasions with fewer than five epochs of sensory-evoked data remaining after artefact rejection were excluded from sensory-evoked brain age estimation (9 out of 138 recordings: for these recordings brain age was estimated using the resting state model alone). Visual and tactile evoked potentials were fitted to neurodynamic response functions based on the characteristic waveforms of the evoked stimulus responses (16) (Supplementary Figures S3-S4 show the average evoked potentials). Zandvoort et al. (2024) extracted these characteristic waveforms using principal component analysis. The visual and tactile responses at channels Oz and Cz, respectively, can be captured by four visual and two tactile neurodynamic response functions (16). Before fitting, evoked responses were Woody filtered to the neurodynamic response functions to improve their temporal alignment, allowing for individual infant differences in the latency of the evoked response (maximum jitter: 50 ms; number of iterations: 1) (37). Neurodynamic response functions were then fitted to the individual's evoked responses using a linear regression. The regression's slope indicates the magnitude of the evoked response. The slopes were obtained for the six neurodynamic response functions and forwarded as input for the sensory brain-age model, which consisted of a support vector regression with a linear kernel function using MATLAB's fitrsvm function (version 2022b; MathWorks). The output of the model is the predicted sensory brain age for the infant [for further details, see Zandvoort et al., 2024 (16)]. The mean absolute error for data presented in this study is comparable to the data presented in the original paper (Supplementary Figure S5).

To calculate the infants' resting state brain age, we used the model developed by Ansari and colleagues (2024) (17). Data were first downsampled to 64 Hz, and the first 20 min of every recording was selected for analysis as this should warrant reliable brain age estimates (17). EEG recordings were started when the infant was settled in their cot/incubator and no overt artefacts were present (data length: 105 ± 41 min [31, 193]; mean ± standard deviation [range]). Since our studies comprised resting-state and stimulus-evoked recordings, we selected a 20-minute window in which no visual or tactile stimuli were applied. This enabled us to standardise the recording length used for analysis across infants. However, in line with Ansari et al. (2024), we show that brain age predictions estimated over 20 min are comparable to predictions from the full recordings (Supplementary Figure S6A) and that using the full recording to estimate the brain age does not alter the relationship between apnoea rate and brain age gap (Supplementary Figure S6B). For one recording, the total data duration was less than 20 min, meaning that we did not have sufficient data to calculate a reliable resting state brain age. For this recording, we only used the brain-age estimate of the sensory model. For all recordings, the bipolar derivative between channels C3 and C4 was used for brain age estimation. The data were input into the deep neural network, and the output of the model was the predicted resting state brain age for the recording [for further details, see Ansari et al. 2024 (17)]. Briefly, this algorithm segments the data into 30-second epochs and estimates a brain age for each of them for ten iterations (the model consisted of a 10-learner ensemble method). To obtain a single brain age value, the median over all ten ensembles was taken for every 30-second epoch, after which a median over all 30-second epochs resulted in a single value. Using the approach of taking a median is robust to the effects of artefacts (17, 38). To validate the output, Ansari et al. (2024) also used an independent test set and found a mean absolute error (MAE) of 1.03 weeks for the held-out dataset. Their model also yielded a significantly lower MAE than a null model estimating the mean PMA of the dataset. Model applications revealed mean absolute errors of 1.22 and 0.77 weeks for the sensory-evoked and resting state models within the data studied here. These accuracies are comparable to those reported in the original studies which were 1.41 and 0.79 weeks. Moreover, for model training, Ansari et al. (2024) used data of infants with normal neurodevelopmental outcome at 24-month follow-up (assessed with Bayley Scale of Infant Development-II). Their model was validated on EEG data that were labelled as normal by a neurophysiologist. Neither brain age models take into account the different sleep states. Whilst it is known that sleep states alter EEG activity in infants (20), derived brain ages are marginally affected by it according to earlier versions of the model (39).

We obtained a single brain age prediction by averaging the brain ages of both the resting-state and sensory models. Since the models may estimate brain age with systematic prediction errors relative to PMA (15), we corrected for this by first fitting a linear model between the combined brain age and PMA. We next predicted PMA from this model and defined the model bias as the difference between the PMA and predicted PMA (meaning that brain age gap and PMA are completely uncorrelated). Next, brain age was corrected by subtracting the bias from the brain age (relative to PMA). This age-bias correction is a standard approach in the brain age field (15, 40). This also ensured that the brain age gap and PMA were uncorrelated. However, without this bias correction, similar results were obtained (Supplementary Figure S7).

#### Probability density functions for the inter-breath intervals

To study how the IBI dynamics changed with PMA and brain age gap, we created probability density functions. To do so, we first created a frequency distribution from the IBIs of each recording at a resolution of 0.1 s. IBIs longer than 50 s were removed from further analysis. Distributions of each recording were normalised so that the area under the curve was equal to 1, allowing for inter-recording comparisons. We next transformed these recording-specific probability density functions into functions specific for a PMA/brain age gap. For this, a weight of 1 or lower was assigned to the recording-specific probabilities, depending on the infant's PMA/brain age gap. Weights were determined using a Gaussian window with a full width at half maximum of 13 days. Weightings for differences of more than 14 days were set to 0. Distributions that were specific to a certain PMA/brain age gap were computed as the weighted median of all distributions together with their weights (41). We created probability density functions between 31 and 36 weeks for the PMA and between −3 and 3 weeks for the brain age gap.

### Statistical analysis

To compare PMA and brain age gap with apnoea rate and respiration rate, we used linear mixed-effects models (computing four models for each of apnoea rate and respiration rate against PMA and brain age gap separately). Previous literature suggested that experiencing apnoeas is inversely associated with gestational age at birth (26, 42). The course of this relationship is, however, unknown which is why we used linear models as a starting point. Nevertheless, we tested the assumption of linearity (see Supplementary Material Table S1) using the mfp package in R. Moreover, it is not known how apnoea rate varies with brain age gap. A linear fit was appropriate for the data collected here so as not to overfit the data; however, whether a linear association underlies the brain age gap/apnoea relationship needs to be studied in future research. Data length and infection were included as confounding factors in the analysis; data length of the IBI recordings and infection (“no”, “suspected”, or “treated”) were included as fixed factors. Infant ID was included as a random factor (to account for the repeated test occasions in the same infant). *P*-values (for the slopes of PMA and brain age gap), partial correlation coefficients and their 95% confidence intervals are reported from these models. The normality assumption was verified with Q-Q plots. Further discussion of the fixed factors to include within the model, and assessment of the results with alternative factors is included in the Supplementary Material. To test for the robustness of the associations and to estimate the strength of unmeasured confounders, we report E-values (43), a widely used method in observational medical studies (44). We provide E-values for the observed association estimate and the confidence interval limit that is closest to null, computed via https://www.evalue-calculator.com. For visualisation, we used the plotAdjustedResponse function in MATLAB to adjust for the fixed factors (calculating a separate model without the random effect of infant). Regression lines are visualised by calculating the line-of-best-fit on the adjusted data.

To test if apnoea rate is better associated with brain age gap than PMA, we first bootstrapped the partial correlation coefficient between apnoea rate and PMA using 10,000 repetitions. This allowed us to generate a 95% confidence interval for the association between apnoea rate and PMA. To test if the partial correlation coefficient between brain age gap and apnoea rate lies outside this interval, the significance value was defined as the number of bootstrapped partial correlation coefficients that were equal to or lower than the true partial correlation coefficient between brain age gap and apnoea rate. Significance was tested one-tailed with an alpha level of 0.05.

For the association between brain age gap and age at caffeine termination, we focused on a subset of 27 infants who had their brain age gap assessed within two weeks before they stopped caffeine treatment (note that infants were all receiving caffeine during the EEG recording). A linear regression was used with infection status as a fixed factor (data length was not included as a fixed factor as all EEG was analysed using a fixed data length, and respiratory data was not analysed for this question). For visualisation, we used the plotAdjustedResponse function adjusting for the fixed effect of infection status only and the regression line was calculated as the line-of-best-fit of the adjusted data.

In an exploratory analysis, we compared brain age gap before infants stopped caffeine treatment with apnoea rate and oxygen desaturation rate in the week after caffeine treatment discontinuation. This consisted of a subset of infants (*n* = 17) with longitudinal recordings of vital signs throughout their hospital stay. These infants were divided into two groups depending on their brain age gap—either mature if their brain age gap >0 weeks or immature if their brain age gap <0 weeks. The brain age gap was calculated from a single EEG recording taken in the two weeks before they stopped caffeine. Apnoeas and desaturations were identified from the recordings in the 7 days after caffeine was stopped. Desaturation events were defined as 10-second periods in which the oxygen saturation was less than 80% (34). The type of vital signs monitoring was dependent on clinical decision, and so 6 out of 17 infants did not have their IP signal measured during the week following caffeine discontinuation (only oxygen saturation and pulse were monitored using a PPG), and they were consequently excluded from the apnoea rate analysis. Due to the limited sample sizes, and as this was an exploratory hypothesis-generating study, we did not apply any statistics and reported outcomes as mean and standard deviation.

## Results and discussion

We included 74 infants on 138 separate occasions (demographic information is provided in Table 1). We included preterm infants between 31 and 36 weeks PMA who were stable and without any significant respiratory or central nervous system complications at the time of the EEG recording (additional details in the Methods). EEG and vital signs were recorded for one to two hours, with continued vital signs recording in some infants up to the time of discharge (see Methods). Sensory-evoked (visual and tactile) and resting state EEG were used to calculate the brain age gap by applying previously developed and validated models (16, 17). We used both sensory-evoked and resting state models to improve accuracy by assessing these different (i.e., sensory-evoked and resting state) aspects of functional brain age gap; results using each model independently are presented in the Supplementary Material and demonstrate similar trends to those presented in the main results (Supplementary Figure S8). In our cohort, the mean brain age gap was 0.01 weeks (standard deviation: 0.91, median: 0.06, interquartile range: 1.08 weeks), with 71 recordings (51%) classed as mature relative to PMA (brain age gap >0) and 67 (49%) classed as immature relative to PMA (brain age gap<0).

**Table 1:** Infant demographics.

Factor	Value
Age	
Gestational age at birth (weeks)	31.0 (23.6–36.6)
Postmenstrual age at recording (weeks)	34.0 (31.0–36.9)
Postnatal age at recording (weeks)	3.6 (0.0–11.4)
Birthweight (g)	1,573 (630–4,525)
Sex	
Females	25/74 (33.8)
Males	49/74 (66.2)
Mode of delivery	
Normal vaginal delivery	13/74 (17.6)
Vaginal breech	2/74 (2.7)
Vaginal assisted (ventouse/forceps/kiwi)	4/74 (5.4)
Elective Caesarean section	8/74 (10.8)
Emergency Caesarean section	47/74 (63.5)
Apgar scores	
Apgar at 1 min	6.9 (1–10)
Apgar at 5 min	8.9 (2–10)
Apgar at 10 min	9.6 (6–10)
Ethnicity	
White	26 (35.1)
Asian	5 (6.8)
Black	1 (1.4)
Mixed	6 (8.1)
Other	1 (1.4)
Information not recorded	35 (47.3)
Resuscitation at birth	
Yes	55/74 (74.3)
No	19/74 (25.7)
Respiratory distress syndrome	
Ongoing condition	47/138 (34.1)
Past treated	57/138 (41.3)
No	34/138 (24.6)
Persistent pulmonary hypertension	
Past treated	4/138 (2.9)
No	134/138 (97.1)
Patent ductus arteriosus	
Ongoing condition	9/138 (6.5)
Past treated	7/138 (5.1)
No	122/138 (88.4)
Jaundice	
Ongoing condition	6/138 (4.3)
Past treated	78/138 (56.5)
No	54/138 (39.1)
Infection	
None	61/138 (44.2)
Suspected	17/138 (12.3)
Treated	60/138 (43.5)
Level of care	
Intensive Therapy Unit	6/138 (4.3)
High Dependency Unit	65/138 (47.1)
Low Dependency Unit	59/138 (42.8)
Postnatal care	5/138 (3.6)
Information not recorded	3/138 (2.2)
Respiratory support	
High flow therapy	35/138 (25.4)
Low flow therapy	14/138 (10.1)
Spontaneous ventilation	88/138 (63.8)
Information not recorded	1/138 (0.7)
Medications received on the day of EEG recording	
Vitamins and minerals	112/138 (81.2)
Probiotics	59/138 (42.8)
Breastmilk fortifier	56/138 40.6)
Caffeine^a^	67/138 (48.6)
Antibiotics	6/138 (4.3)
Gaviscon	6/138 (4.3)
Paracetamol	2/138 (1.4)
Budesonide	6/138 (4.3)
Nystatin	1/138 (0.7)
Diuretics	1/138 (0.7)
No medication	16/138 (11.6)

Reported values are mean (range) or number (%). Antibiotics include: benzylpenicillin, gentamicin, coamoxiclav, cefotaxime, chloramphenicol, and amoxicillin; vitamins and minerals include: abidec, calcium, phosphate, vitamin D, folic acid, and iron; diuretics include: chlorothiazide and spironolactone.

a For those infants who received caffeine at the time of EEG recording, the dose was 5 mg/kg for 57 infants, 7.5 mg/kg for 6 infants and 10 mg/kg for 4 infants.

**Figure 2:**
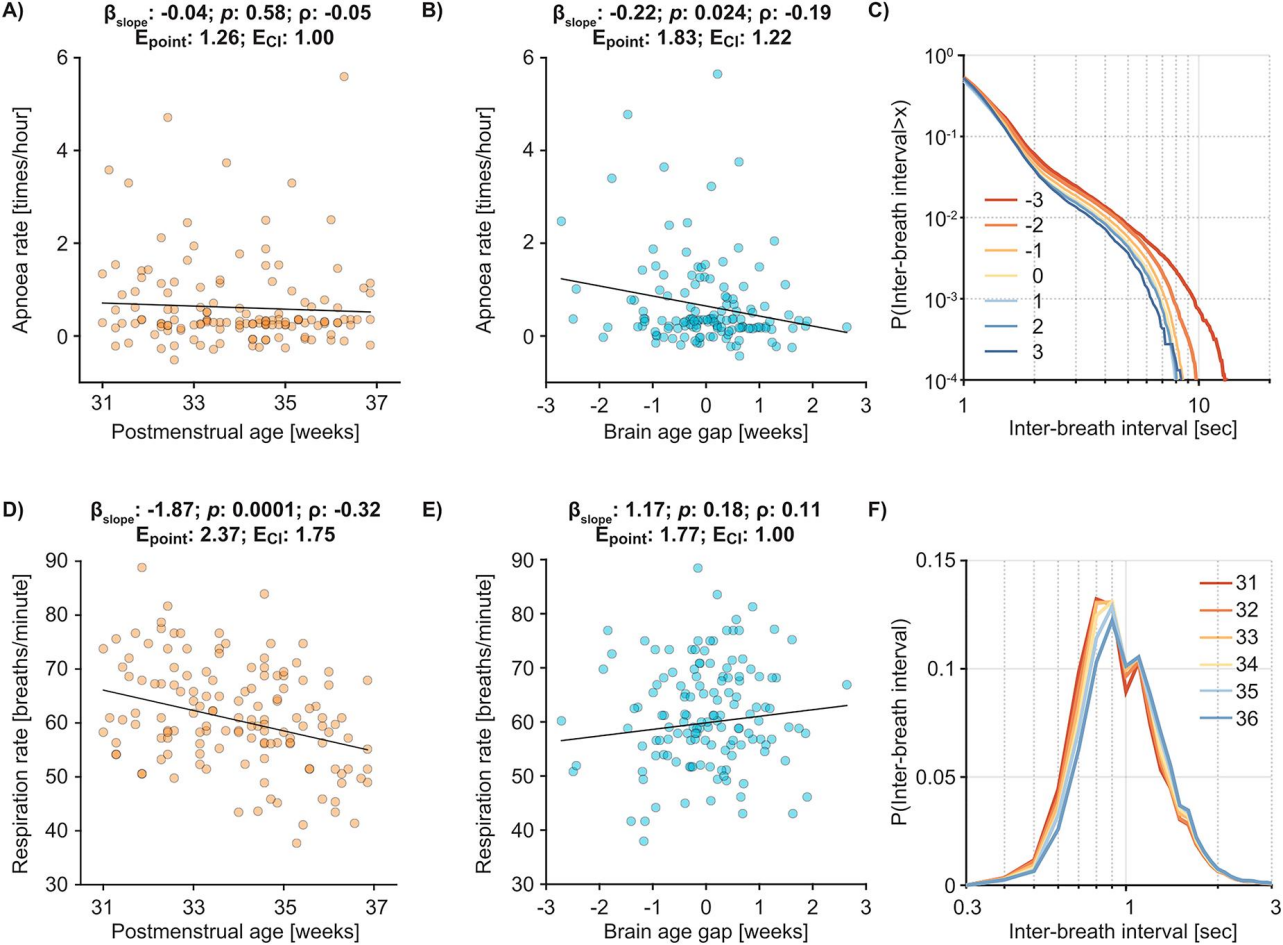
Apnoea rate is related to brain age gap. The relationship between apnoea rate and **(A)** postmenstrual age (PMA), and **(B)** brain age gap. Each dot indicates an individual test occasion with 74 infants included on 138 test occasions for all panels. Black line is the mean fit of the linear model (*β*_slope_, *p*, *ρ*, E_point_, and E_CI_ in the titles are the predictor's regression slope, and its significance, partial correlation coefficient, E-value point estimate, and E-value of the confidence interval, respectively). **(C)** The cumulative probability distribution of inter-breath intervals split according to brain age gap (indicated by different colours from −3 up to 3 weeks). The distribution is shown on a double logarithmic scale to emphasise the prolonged inter-breath intervals (i.e., apnoeas). **(D,E)** The relationship between respiratory rate and **(D)** PMA and **(E)** brain age gap. **(F)** The probability density distributions of inter-breath intervals shown according to the infant's PMA. Statistical models in panels A and B are adjusted for data length of impedance pneumograph (i.e., the signal used to assess apnoea rate) and infection; panels D and E are adjusted for infection (see Supplementary Results for discussion of statistical models and Supplementary Figures S10-S16).

Apnoea rate was not significantly associated with PMA in the age range studied (*p*:0.58; *β* [95% CI]:−0.04 [−0.16 to 0.09]; *ρ*:−0.0472; E_point_:1.26; E_CI_:1.00; Figure 2A). Whilst we would expect that apnoea rate decreases with PMA when considering a wider age range (6), this result demonstrates that apnoea rate is not strongly related to PMA in older preterm infants, highlighting potential pitfalls of guidelines based on age for late preterm infants. Consistent with our hypothesis, apnoea rate was significantly related to brain age gap (*p*:0.024; *β* [95% CI]:−0.22 [−0.41 to −0.03]; *ρ*:−0.19; E_point_:1.83; E_CI_:1.22; Figure 2B, comparison of the relationship between apnoea rate and brain age gap vs. apnoea rate and PMA: *p*:0.12; one-tailed bootstrapped 95% CI: [−0.2287 to 1]). There was a clear relationship between brain age gap and the distribution of prolonged inter-breath intervals, with immature brain activity associated with longer inter-breath intervals (Figure 2C, infants with immature brain age gap relative to PMA—indicated by negative values and shown in the figure with red/orange colours—have a distribution shifted to the right of those with mature brain age gap [shown in blue], i.e., higher apnoea rates for different IBI thresholds occur in infants with immature brain activity). This suggests the association between apnoea rate and brain age gap holds irrespective of the exact definition of apnoea used, which is variable within clinical practice (45). This relationship between apnoea rate and brain age gap was also observed in the subgroup of infants receiving caffeine at the time of study (Supplementary Figure S9), providing preliminary indications of the potential value of brain age gap as a candidate biomarker for caffeine requirement. It is important to note that our method for assessing respiration (impedance pneumography) is limited to the detection of central apnoeas; we did not measure obstructive apnoeas. Central apnoeas are predominantly related to immaturity of the nervous system. In preterm infants, the development of central chemoreceptors is immature, and there is a blunted central nervous system response to hypercapnia (42). This immaturity of respiratory centres, which we postulate is related to general immaturity of the brain as assessed here, likely underlies these results. Moreover, these results may be related to immaturity of the cortical control of respiration (27).

Interestingly, when considering the average respiratory rate (i.e., normal breathing between apnoeas) we observed the opposite results—respiratory rate significantly decreased with PMA (*p*:<0.001; *β* [95% CI]:−1.87 [−2.81 to −0.93]; *ρ*:−0.3186; E_point_:2.37; E_CI_: 1.75; Figures 2D–F) and respiratory rate did not significantly relate to brain age gap (*p*:0.18; *β* [95% CI]:1.17 [−0.54 to 2.88]; *ρ*:0.1149; E_point_:1.77; E_CI_: 1.00; Figure 2E). After birth, critical changes in lung and chest wall dynamics play a major role in developing breathing mechanics (46). With advancing PMA, lung alveoli mature both functionally and anatomically, improving lung-chest wall elasticity (47). We speculate that these changes play a major role in driving decreases in respiratory rate with advancing PMA. The contrasting results between apnoea and respiratory rate underlie the distinction between normal breathing and apnoea and further highlight the importance of utilising brain age gap as a biomarker for caffeine requirement in preterm infants.

**Figure 3:**
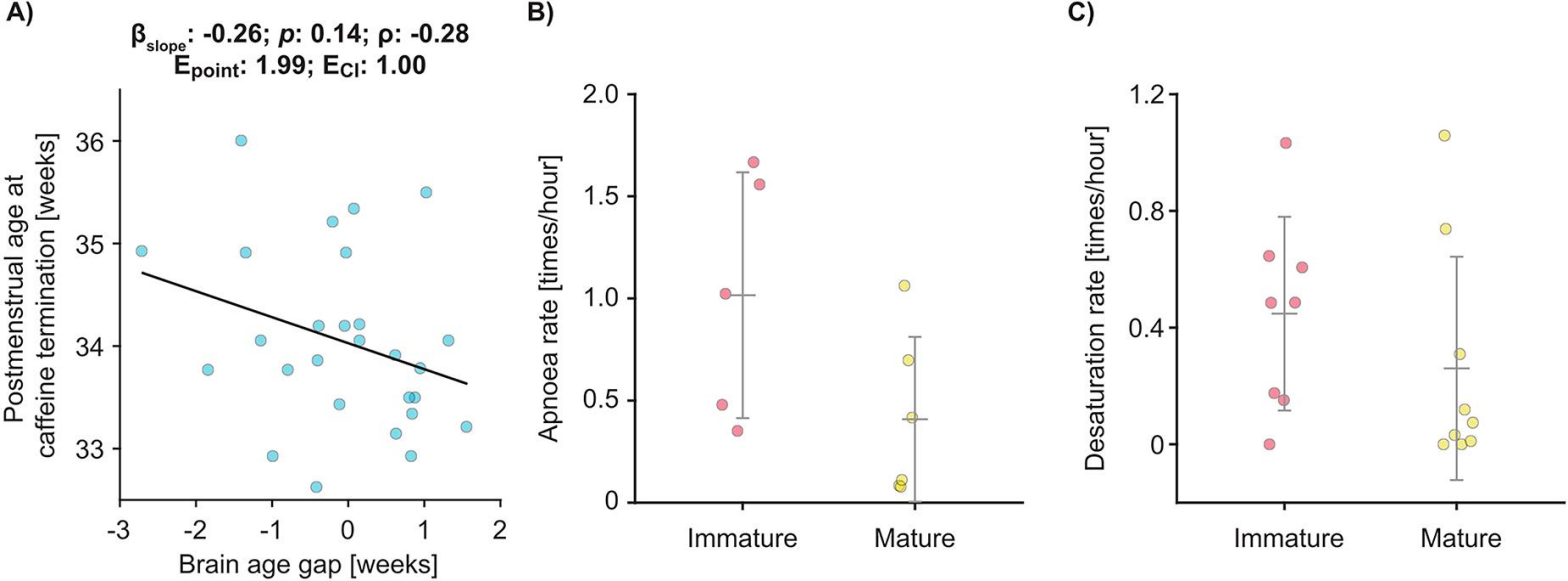
Caffeine requirement may be related to brain age gap. **(A)** The relationship between age at which caffeine treatment is discontinued compared to brain age gap in the subset of 27 infants where brain activity was recorded in the two weeks before caffeine was discontinued. Each dot indicates an infant. Black line is the mean fit of the linear model (*β*_slope_, *p*, *ρ*, E_point_, and E_CI_ in the title is the predictor's regression slope, and its significance, partial correlation coefficient, E-value point estimate, and E-value of the confidence interval, respectively). **(B,C)** Exploratory hypothesis-generating study in the subset of infants where vital signs were recorded longitudinally. Infants were grouped according to whether their brain activity was immature or mature relative to their PMA before they discontinued caffeine treatment. Error bars indicate mean and standard deviation. **(B)** Apnoea rate (*n* = 11 infants) and **(C)** desaturation rate (*n* = 17 infants) in the 7 days after caffeine treatment was stopped.

Guidelines for discontinuing caffeine treatment are related to PMA, but there is variation in when treatment is stopped (48), depending on clinical discretion and guided by the infant's physiological stability. Given that apnoea rate is related to brain age gap, we hypothesised that the age at which infants stopped caffeine treatment would be related to their brain age gap. We explored this in a subset of 27 infants who had their EEG recorded shortly before discontinuing caffeine treatment (decision to stop caffeine was at clinicians discretion, clinicians were blinded to EEG recording outcomes, time between EEG and caffeine discontinuation was a median of 4 days (IQR: 2−7), with a range of 0–13 days). Note all infants stop caffeine whilst in the neonatal unit, however, for most infants we did not record their EEG at the time they stopped caffeine treatment, and so they were not included in this subset analysis. In this subset, we found that the age at which infants stopped caffeine treatment was correlated with their brain age gap (*p*:0.14; *β* [95% CI]:−0.26 [−0.61 to 0.09]; *ρ*:−0.2839; E_point_:1.99; E_CI_:1.00; Figure 3A) with infants with immature brain activity discontinuing caffeine at older ages. However, despite this association, some infants with relatively mature brain activity continued caffeine close to term-corrected age, and, *vice versa*, some infants with immature brain activity discontinued treatment at a much younger age. Using an infant's brain age gap will provide an objective metric to identify when to discontinue treatment.

Finally, in an exploratory hypothesis-generating study in a subset of infants who had longitudinal recordings of vital signs throughout their hospital stay (see Methods), we investigated whether there was a relationship between brain age gap (recorded before infants discontinued caffeine, the time between EEG recording and caffeine discontinuation was a median of 4 days (IQR: 3–7), with a range of 0–13 days), and apnoea rate in the 7 days after they discontinued caffeine treatment. Infants with more mature brain activity (defined as brain age gap >0) had 60% fewer apnoeas and 42% fewer oxygen desaturations after they stopped caffeine treatment compared with those infants with immature brain activity (brain age gap <0) (apnoea rate, *n* = 11: infants with mature brain activity: 0.41 ± 0.40 apnoeas/hour [mean ± SD]; infants with immature brain activity: 1.02 ± 0.60; Figure 3B; desaturations *n* = 17: infants with mature brain activity: 0.26 ± 0.38 desaturations/hour; infants with immature brain activity: 0.45 ± 0.33; Figure 3C). As this exploratory study was limited in sample size and conducted to determine if there was any preliminary evidence of a relationship between brain age gap and apnoea rate after caffeine discontinuation, no statistical analyses were performed. Another limitation is that this was a single-centre study comprising mainly Caucasian babies (Table 1). The study did not follow a pre-registered protocol and only examined the subset of infants that had longitudinal recordings at the time of stopping caffeine. These results should therefore be replicated in a prospective study before definitive conclusions can be drawn. Indeed, a prospective study is first needed to ascertain whether brain age gap at the exact time of stopping caffeine is related to apnoea rate; here the exploratory nature meant that the EEG recording was within 2 weeks before caffeine was stopped—it is conceivable that brain age gap changed in this time. Moreover, external validation would be essential to use brain age gap clinically. Validation studies would be required to assess applicability of these measures across different populations (for example, with different ethnicities, co-morbidities and gestational age at birth), with different EEG recording systems, in different healthcare settings and to compare with other centre-specific caffeine discontinuation practices. Nevertheless, our findings provide promising initial evidence that physiological instability after discontinuation of caffeine is related to brain age gap.

The results presented here demonstrate that apnoea rate is related to brain age gap, not postmenstrual age, in infants from 31 to 36 weeks. We focused on this age range as this is the age at which doctors will consider whether or not infants require caffeine treatment, and unlike at younger ages, not all infants will experience apnoea. In infants of this age range who are experiencing apnoea and/or desaturations, it can be difficult to disentangle the cause, which may be brain immaturity but also other factors such as infection or feeding problems—for which caffeine is not the best course of treatment. We propose that brain age gap (once further validated in other studies) is a candidate biomarker which could be used as an objective approach to dissociate the underlying mechanism of apnoea and determine whether it is appropriate to stop caffeine treatment. Future work should also assess whether EEG is a candidate biomarker for caffeine treatment in younger infants. Whilst all extremely preterm infants are likely to need caffeine at birth, it is plausible that brain age gap may be a useful candidate biomarker to indicate the dose of caffeine an individual infant will require.

We identified a significant relationship between apnoea rate and brain age gap, nevertheless, there was heterogeneity in responses. Within the statistical model, we adjusted for data length and the presence of infection, and additionally mode of ventilation (see Supplementary Results). We did not adjust for gestational age or postnatal age, as in our sample this was highly colinear with PMA (see Supplementary Results). Future work to investigate heterogeneity in responses is required, including further understanding of the impact of clinical and demographic factors, such as gestational age at birth, co-morbidities and medication (such as caffeine), on both brain age gap itself and the relationship between apnoea rate and brain age gap.

Finally, further work is needed to translate the results presented here into a clinically usable tool: EEG is already used in neonatal units, however, technology to incorporate brain age assessment within an EEG device is needed. The recently developed NeoNaid software is a step towards this, providing brain age (and sleep state) from EEG in a user-friendly interface which includes quality control metrics enabling clinicians to check that the data they are acquiring are suitable for brain age estimation (38). The reduced EEG montages and required length of recording for recent brain age models also means this technique is more feasible in the clinical setting (17, 38). Nevertheless, further studies investigating test-retest reliability and clinical implementation in different neonatal settings, potentially integrating these metrics into standard neonatal equipment, are necessary. Additionally, prospective studies evaluating the relationship between brain age at the time of stopping caffeine therapy and apnoea rate after stopping caffeine treatment are needed. Such studies could be used to build a model to identify the optimal brain age at which to stop caffeine treatment in premature infants. The derived model could then be integrated into a clinical decision support system, providing real-time insight for clinicians regarding the decision to stop caffeine. Such a model/clinical decision support system would then need to be assessed in a prospective randomised controlled trial which would assess both the efficacy and safety of using the clinical decision support system compared with standard care. Indeed, a limitation of the work here is that we do not provide clinical decision modelling or predictive performance metrics for stopping caffeine therapy based on different brain ages. This was outside the scope of the study, which was exploratory and hypothesis-generating, and specifically aimed to investigate the relationship between apnoea rate and brain age gap. Prospective validation studies are essential before brain age gap could be used clinically. Nevertheless, the current work provides the foundations to suggest that such a model, which uses brain age gap as a candidate biomarker, has strong potential.

## Conclusions

In summary, we demonstrate that apnoea rate is related to brain age gap, not PMA, in infants from 31 to 36 weeks PMA. We also provide promising initial evidence that brain age gap is related to apnoea rate and oxygen desaturations in the week after caffeine is stopped. Whilst the utilisation of brain age as a biomarker for caffeine requirement requires much additional validation, our current study provides a foundation for its use. There is a need for personalised medicine in neonatology; this study demonstrates that brain age gap is a candidate biomarker for tailored caffeine treatment.

## Data Availability

The raw data supporting the conclusions of this article will be made available by the authors, without undue reservation.
